# Nucleoside reverse-transcriptase inhibitor cross-resistance and outcomes from second-line antiretroviral therapy in the public health approach: an observational analysis within the randomised, open-label, EARNEST trial

**DOI:** 10.1016/S2352-3018(17)30065-6

**Published:** 2017-05-08

**Authors:** Nicholas I Paton, Cissy Kityo, Jennifer Thompson, Immaculate Nankya, Leonard Bagenda, Anne Hoppe, James Hakim, Andrew Kambugu, Joep J van Oosterhout, Mary Kiconco, Silvia Bertagnolio, Philippa J Easterbrook, Peter Mugyenyi, A Sarah Walker, Europe Africa Research Network for Evaluation of Second-line Therapy (EARNEST) Trial Team, E Agweng, P Awio, G Bakeinyaga, C Isabirye, U Kabuga, S Kasuswa, M Katuramu, C Kityo, F Kiweewa, H Kyomugisha, E Lutalo, P Mugyenyi, D Mulima, H Musana, G Musitwa, V Musiime, M Ndigendawan, H Namata, J Nkalubo, P Ocitti Labejja, P Okello, P Olal, G Pimundu, P Segonga, F Ssali, Z Tamale, D Tumukunde, W Namala, R Byaruhanga, J Kayiwa, J Tukamushaba, S Abunyang, D Eram, O Denis, R Lwalanda, L Mugarura, J Namusanje, I Nankya, E Ndashimye, E Nabulime, D Mulima, O Senfuma, G Bihabwa, E Buluma, P Easterbrook, A Elbireer, A Kambugu, D Kamya, M Katwere, R Kiggundu, C Komujuni, E Laker, E Lubwama, I Mambule, J Matovu, A Nakajubi, J Nakku, R Nalumenya, L Namuyimbwa, F Semitala, B Wandera, J Wanyama, H Mugerwa, A Lugemwa, E Ninsiima, T Ssenkindu, S Mwebe, L Atwine, H William, C Katemba, S Abunyang, M Acaku, P Ssebutinde, H Kitizo, J Kukundakwe, M Naluguza, K Ssegawa, Namayanja, F Nsibuka, P Tuhirirwe, M Fortunate, J Acen, J Achidri, A Amone, M Chamai, J Ditai, M Kemigisa, M Kiconco, C Matama, D Mbanza, F Nambaziira, M Owor Odoi, A Rweyora, G Tumwebaze, H Kalanzi, J Katabaazi, A Kiyingi, M Mbidde, M Mugenyi, R Mwebaze, P Okong, I Senoga, M Abwola, D Baliruno, J Bwomezi, A Kasede, M Mudoola, R Namisi, F Ssennono, S Tuhirwe, G Abongomera, G Amone, J Abach, I Aciro, B Arach, P Kidega, J Omongin, E Ocung, W Odong, A Philliam, H Alima, B Ahimbisibwe, E Atuhaire, F Atukunda, G Bekusike, A Bulegyeya, D Kahatano, S Kamukama, J Kyoshabire, A Nassali, A Mbonye, T M Naturinda, Ndukukire, A Nshabohurira, H Ntawiha, A Rogers, M Tibyasa, S Kiirya, D Atwongyeire, A Nankya, C Draleku, D Nakiboneka, D Odoch, L Lakidi, R Ruganda, R Abiriga, M Mulindwa, F Balmoi, S Kafuma, E Moriku, J Hakim, A Reid, E Chidziva, G Musoro, C Warambwa, G Tinago, S Mutsai, M Phiri, S Mudzingwa, T Bafana, V Masore, C Moyo, R Nhema, S Chitongo, Robert Heyderman, Lucky Kabanga, Symon Kaunda, Aubrey Kudzala, Linly Lifa, Jane Mallewa, Mike Moore, Chrissie Mtali, George Musowa, Grace Mwimaniwa, Rosemary Sikwese, Joep van Oosterhout, Milton Ziwoya, H Chimbaka, B Chitete, S Kamanga, T Kayinga E Makwakwa, R Mbiya, M Mlenga, T Mphande, C Mtika, G Mushani, O Ndhlovu, M Ngonga, I Nkhana, R Nyirenda, P Cheruiyot, C Kwobah, W Lokitala Ekiru, M Mokaya, A Mudogo, A Nzioka, A Siika, M Tanui, S Wachira, K Wools-Kaloustian, P Alipalli, E Chikatula, J Kipaila, I Kunda, S Lakhi, J Malama, W Mufwambi, L Mulenga, P Mwaba, E Mwamba, A Mweemba, M Namfukwe, E Kerukadho, B Ngwatu, J Birungi, N Paton, J Boles, A Burke, L Castle, S Ghuman, L Kendall, A Hoppe, S Tebbs, M Thomason, J Thompson, S Walker, J Whittle, H Wilkes, N Young, M Spyer, C Kapuya, F Kyomuhendo, D Kyakundi, N Mkandawire, S Mulambo, S Senyonjo, B Angus, A Arenas-Pinto, A Palfreeman, F Post, D Ishola, J Arribas, R Colebunders, M Floridia, M Giuliano, P Mallon, P Walsh, M De Rosa, E Rinaldi, I Weller, C Gilks, J Hakim, A Kangewende, S Lakhi, E Luyirika, F Miiro, P Mwamba, P Mugyenyi, S Ojoo, N Paton, S Phiri, J van Oosterhout, A Siika, S Walker, A Wapakabulo, T Peto, N French, J Matenga, G Cloherty, J van Wyk, M Norton, S Lehrman, P Lamba, K Malik, J Rooney, W Snowden, J Villacian

**Affiliations:** a Yong Loo Lin School of Medicine, National University of Singapore, Singapore; b Joint Clinical Research Centre (JCRC), Kampala, Uganda; c MRC Clinical Trials Unit at University College London, London, UK; d University of Zimbabwe Clinical Research Centre, Harare, Zimbabwe; e Infectious Diseases Institute, Kampala, Uganda; f Department of Medicine, University of Malawi College of Medicine, Blantyre, Malawi; g Dignitas International, Zomba, Malawi; h JCRC, Fort Portal, Uganda; i World Health Organization, Geneva, Switzerland

## Abstract

**Background:**

Cross-resistance after first-line antiretroviral therapy (ART) failure is expected to impair activity of nucleoside reverse-transcriptase inhibitors (NRTIs) in second-line therapy for patients with HIV, but evidence for the effect of cross-resistance on virological outcomes is limited. We aimed to assess the association between the activity, predicted by resistance testing, of the NRTIs used in second-line therapy and treatment outcomes for patients infected with HIV.

**Methods:**

We did an observational analysis of additional data from a published open-label, randomised trial of second-line ART (EARNEST) in sub-Saharan Africa. 1277 adults or adolescents infected with HIV in whom first-line ART had failed (assessed by WHO criteria with virological confirmation) were randomly assigned to a boosted protease inhibitor (standardised to ritonavir-boosted lopinavir) with two to three NRTIs (clinician-selected, without resistance testing); or with raltegravir; or alone as protease inhibitor monotherapy (discontinued after week 96). We tested genotypic resistance on stored baseline samples in patients in the protease inhibitor and NRTI group and calculated the predicted activity of prescribed second-line NRTIs. We measured viral load in stored samples for all patients obtained every 12–16 weeks. This trial is registered with Controlled-Trials.com (number ISRCTN 37737787) and ClinicalTrials.gov (number NCT00988039).

**Findings:**

Baseline genotypes were available in 391 (92%) of 426 patients in the protease inhibitor and NRTI group. 176 (89%) of 198 patients prescribed a protease inhibitor with no predicted-active NRTIs had viral suppression (viral load <400 copies per mL) at week 144, compared with 312 (81%) of 383 patients in the protease inhibitor and raltegravir group at week 144 (p=0·02) and 233 (61%) of 280 patients in the protease inhibitor monotherapy group at week 96 (p<0·0001). Compared with results with no active NRTIs, 95 (85%) of 112 patients with one predicted-active NRTI had viral suppression (p=0·3) and 20 (77%) of 26 patients with two or three active NRTIs had viral suppression (p=0·08). Over all follow-up, greater predicted NRTI activity was associated with worse viral load suppression (global p=0·0004).

**Interpretation:**

Genotypic resistance testing might not accurately predict NRTI activity in protease inhibitor-based second-line ART. Our results do not support the introduction of routine resistance testing in ART programmes in low-income settings for the purpose of selecting second-line NRTIs.

**Funding:**

European and Developing Countries Clinical Trials Partnership, UK Medical Research Council, Institito de Salud Carlos III, Irish Aid, Swedish International Development Cooperation Agency, Instituto Superiore di Sanita, WHO, Merck.

## Introduction

More than 17 million people receive antiretroviral therapy (ART) for HIV infection, mainly delivered with the WHO-recommended public health approach, characterised by use of standardised sequential regimens.^1^, ^2^ Standardised first-line and second-line regimens both include nucleoside reverse-transcriptase inhibitors (NRTIs), combined with a non-NRTI (NNRTI) in first-line and a boosted protease inhibitor in second-line therapy.^3^ Treatment guidelines for individualised therapy in high-income settings generally recommend that resistance testing be done at the time of first-line failure, because of the assumption that choosing more active NRTI drugs (those with less predicted cross-resistance) will probably optimise outcomes.^4^, ^5^, ^6^ However, in programmes that have adopted the public health approach, in which patients often have prolonged exposure to a failing first-line regimen (because diagnosis of failure is delayed by limited access to viral load monitoring) with extensive cross-resistance to second-line NRTIs (which is usually undetected, because resistance testing is not done routinely), treatment outcomes seem excellent.^7^, ^8^ This suggests that, in the setting of protease inhibitor-based second-line therapy, greater cross-resistance and lower predicted activity of NRTIs do not greatly affect outcomes. Such a scenario might reduce the potential benefit that could be gained from the introduction of resistance testing into the public health approach for second-line NRTI drug selection. We aimed to assess the association between the activity, predicted by resistance testing, of the NRTIs used in second-line therapy and treatment outcomes for patients with HIV.

**Research in context**

**Evidence before this study**

We searched PubMed with no start date or language restrictions for articles published until Aug 1, 2016, with the terms “second-line therapy”, “protease inhibitors”, “resistance testing”, and the individual drug names, and we also reviewed relevant HIV conference abstracts. This search identified studies that examined the association between nucleoside reverse-transcriptase inhibitor (NRTI) resistance and virological outcomes for NRTI plus protease inhibitor second-line therapy after failure on a first-line non-NRTI (NNRTI)-containing regimen. We found two cohort studies done in sub-Saharan Africa (243 and 101 participants, follow-up 12 months) and one in Asia (105 participants, follow-up 12 months) and analyses of the NRTI plus protease inhibitor arm in two randomised controlled trials (254 participants, follow-up 48 weeks; 270 participants, follow-up 96 weeks). These studies either reported no association or an inverse association between predicted activity of prescribed NRTIs and viral load suppression.

**Added value of this study**

In the EARNEST trial, we prospectively followed up a large group of HIV-infected patients taking a protease inhibitor and NRTI second-line regimen and collected adherence data and relevant outcome data, including viral load, with greater frequency and with lower incidence of loss-to-follow-up than in preceding cohort studies. This trial was also larger (426 patients on protease inhibitor and NRTI) than the other two randomised controlled trials of second-line therapy and had longer follow-up (144 weeks). Our analysis shows that a regimen containing NRTIs with no or limited predicted activity achieved viral load suppression in a high proportion of patients, which was sustained throughout longer-term follow-up. The size and duration of our study also allows for a more robust model for analysing predictors of outcome than previous studies: we found a paradoxical inverse association between extent of baseline resistance and viral suppression (noted in several, but not all, previous analyses) but were also able to show more clearly that this inverse association persisted after adjustment for other relevant factors including baseline viral load and CD4 cell count and adherence during follow-up (adherence was proposed as the probable explanation for the inverse association by several previous studies). The main added value of this analysis is the availability of two comparison groups within the same trial, allowing us to show the contribution of the NRTIs to regimen activity. In the group with no predicted active NRTIs in the prescribed regimen, viral load suppression was at least as good as that of a regimen containing a predicted fully active drug from a new drug class (raltegravir) and clearly superior to that obtained with the protease inhibitor used alone. Thus the good outcomes obtained with a protease inhibitor and NRTI second-line regimen in programme settings are not solely attributable to the high potency of protease inhibitors (proposed as the explanation in some earlier studies) but also reflect a substantial additional contribution from NRTIs predicted to have limited or no activity as a result of cross-resistance.

**Implications of all the available evidence**

Despite extensive cross-resistance, NRTIs make a major contribution to viral load suppression in second-line therapy when combined with a protease inhibitor. Predictions based on genotypic resistance testing appear to overestimate the detrimental effect of resistance mutations on the activity of NRTIs and should be re-examined for use in the context of second-line switch to a protease inhibitor-based regimen and possibly for other specific regimen changes. Taken together, the data support WHO treatment guidelines that do not recommend resistance testing at switch to second-line therapy. Adherence seems to be an important determinant of outcomes in second-line therapy and NRTI selection might best be based on minimising toxicity and maximising tolerability, in view of the effect that these might have on adherence.

## Methods

### Study design and patients

We did an observational analysis within an open-label, randomised trial of second-line ART (EARNEST) in sub-Saharan Africa. The EARNEST trial,^7^ done from 2010–14 in 14 clinical sites in Uganda, Zimbabwe, Malawi, Kenya, and Zambia, enrolled adults and adolescents older than 12 years infected with HIV in whom first-line NNRTI-based regimens had failed (by WHO clinical, immunological, or virological criteria, all confirmed by viral load >400 copies per mL; and with no more than three missed ART doses reported in the previous 1 month). The EARNEST trial protocol (including viral load and genotyping sub-studies described here) was approved by ethics committees in participating countries and the UK. All patients or caregivers provided written informed consent.

Eligible patients were randomly assigned using computer-generated randomisation list with variable block size to receive a protease inhibitor (standardised to ritonavir-boosted lopinavir) together with two or three NRTIs (protease inhibitor and NRTI group), or with raltegravir (protease inhibitor and raltegravir group), or alone as monotherapy (following an initial induction with 12 weeks' raltegravir; protease inhibitor monotherapy group). In the protease inhibitor and NRTI group, NRTIs were selected by clinicians (without resistance testing) on the basis of NRTIs used first-line, side-effects, local standard-of-care, drug availability, and WHO guidelines (tenofovir and lamivudine or emtricitabine used if zidovudine or stavudine was used in first-line; zidovudine and lamivudine used if tenofovir was used in first-line).

### Procedures

Patients were followed for 144 weeks with clinic visits every 1–2 months. Adherence was assessed by structured questions and intensive adherence counselling was given when lapses were identified. Treatment was monitored clinically and with CD4 cell counts every 12–16 weeks.

We measured viral load on stored samples in a central laboratory (JCRC, Kampala, Uganda) with the Abbott RealTime HIV-1 assay (Abbott Laboratories, Chicago, IL, USA) at weeks 4, 12, 24, 36, 48, 60, 80, 96, 110, 126 and 144. In the protease inhibitor monotherapy group, viral load was tested at all timepoints to week 48, and at week 96, after which the group was discontinued. Viral load results were reviewed by a data monitoring committee, but not returned to managing physicians.^7^

We genotyped reverse transcriptase on all available baseline samples in the protease inhibitor and NRTI group using a WHO-accredited in-house assay^9^ at JCRC. We used the Stanford algorithm (version 7) to predict drug susceptibility.

We calculated the number of predicted-active NRTIs in the prescribed second-line regimen by counting the NRTI drugs initially prescribed that had no more than low-level resistance (ie, not intermediate or high level resistance) predicted from baseline genotyping. We also calculated the genotypic susceptibility score^10^ for the prescribed second-line NRTIs by assigning a score of 0 (high-level resistance), 0·25 (intermediate resistance), 0·50 (low-level resistance), 0·75 (potential low-level resistance), and 1 (susceptible) to each prescribed NRTI.

### Statistical analysis

Analyses were by intention-to-treat (ie, including patients regardless of subsequent treatment changes) and based on observed viral loads (ie, not imputing missing viral load data). We used generalised estimating equations (independent working correlation structure, binomial distribution, and robust variance) to test differences between treatment groups in the proportion of patients with virological suppression across the whole follow-up period. Risk differences were used to compare groups at the latest timepoint in the study (week 144; or week 96 for the protease inhibitor monotherapy group because this group was discontinued early). We used logistic multivariable regression with fractional polynomials and backwards elimination (exit p>0·05, stata command mfp) to identify independent predictors of a viral load of less than 400 copies per mL at week 144 in complete cases in the protease inhibitor and NRTI group. Variables included in the analysis were age (continuous), sex, viral subtype, proportion of visits missed or when non-adherence was self-reported (continuous), years on first-line ART (continuous), history of ever smoking, ever drinking alcohol, years of education, job status (employed, student, or unemployed), hours worked if employed (continuous), household income (<US$50, $50–200, or >$200 per month), availability of food for regular meals, clinical history (diabetes, tuberculosis, CNS disease, cardiovascular disease), and, at switch to second-line, viral load, CD4 cell count, haemoglobin, blood glucose, and estimated glomerular filtration rate (eGFR; all continuous). We tested the additional effect of individual mutations, pairs of mutations with more than 10% prevalence, and the presence of mutations in the well recognised thymidine analogue mutations type 1 (TAM1; ie, 41Leu, 210Trp, or 215Tyr) and type 2 (TAM2; ie, 67Asn, 70Arg, 215Phe, or 219Gln) pathways one at a time in the selected model. We also did two sensitivity analyses, first without backwards elimination including all factors with p<0·2 on univariable analysis (appendix p 6–7), and second with backwards elimination with exit p>0·2. We used Stata (version 13.1) for all analyses.

### Role of the funding source

The funders of the study had no role in study design, data collection, data analysis, data interpretation, or writing of the report. The corresponding author had full access to all the data in the study and had final responsibility for the decision to submit for publication.

## Results

Between April 12, 2010, and April 29, 2011, 1277 patients were randomly assigned (426 to protease inhibitor and NRTI, 433 to protease inhibitor and raltegravir, 418 to protease inhibitor monotherapy). Baseline characteristics were similar between the groups (42% had viral load >100 000 copies per mL, 62% had CD4 cell count <100 cells per mL).^7^ At 144 weeks, 106 (8%) had died and 30 (2%) had withdrawn or were lost to follow-up. The protease inhibitor monotherapy group changed to combination therapy (mostly adding NRTIs) following review of the week 96 results by the data monitoring committee; two participants in the protease inhibitor and NRTI group switched for failure by week 144; no patients in the protease inhibitor and raltegravir group switched for failure. Viral load results were available from 12 327 (91%) of 13 481 scheduled follow-up timepoints until death, last follow-up, or lost to follow-up.

Baseline genotypes were available for 391 (92%) of 426 participants in the protease inhibitor and NRTI group. 95% had at least one NRTI mutation (table 1). 230 patients (59%) had no predicted-active NRTIs in their prescribed second-line regimen: regimen included tenofovir plus lamivudine or emtricitabine in 176 (77%); patients had median five NRTI mutations; 75% with TAM1 and 69% with TAM2. 128 (33%) of 391 had one predicted-active NRTI prescribed: regimen included tenofovir plus lamivudine or emtricitabine in 113 (88%); median two mutations; 30% with TAM1 and 47% with TAM2. 33 (8%) of 391 had at least two predicted-active NRTIs prescribed: regimen included tenofovir plus lamivudine or emtricitabine in 26 (79%); median zero mutations.

**Table 1:** Resistance at first-line failure and predicted activity of NRTIs prescribed in second-line protease inhibitor and NRTI regimen

		**Total**	**Number of predicted-active NRTIs in second-line regimen**		
			0	1	≥2
Baseline genotype results available		391	230	128	33
GSS of prescribed NRTIs		0·25 (0–1; 0–3)	0 (0–0·25; 0–0·5)	1 (0·5–1; 0·5–1·25)	2 (2–2; 1–3)
Country					
	Malawi*	37 (9%)	17 (7%)	8 (6%)	12 (36%)
	Uganda	253 (65%)	154 (67%)	87 (68%)	12 (36%)
	Zimbabwe	84 (21%)	56 (24%)	21 (16%)	7 (21%)
	Kenya	17 (4%)	3 (1%)	12 (9%)	2 (6%)
Age (years)		37 (31–43)	37 (31–43)	37 (31–45)	36 (24–41)
Years on first line ART		4·0 (2·8–5·4)	4·1 (2·9–5·5)	4·2 (2·7–5·3)	2·8 (2·3–5·0)
Viral load (copies per mL)		68 359 (23 200–181 887)	83 634 (34 800–222 514)	47 733 (13 683–94 293)	113 982 (10 683–325 122)
	>100 000	158 (40%)	110 (48%)	29 (23%)	19 (58%)
CD4 (cells per μL)		69 (28–136)	47 (21–98)	116 (64–183)	91 (50–177)
	≤100	247 (63%)	174 (76%)	54 (42%)	19 (58%)
Number of NRTI mutations		4 (2–5)	5 (4–5)	2 (2–3)	0 (0–1)
Any NRTI mutation		370 (95%)	230 (100%)	128 (100%)	12 (36%)
Any TAM1 mutations		211 (54%)	173 (75%)	38 (30%)	0
Any TAM2 mutations		220 (56%)	159 (69%)	60 (47%)	1 (3%)
Most common NRTI mutations					
	Met184Val	355 (91%)	217 (94%)	126 (98%)	12 (36%)
	Met41Leu	170 (43%)	158 (69%)	12 (9%)	0
	Thr215Tyr	167 (43%)	137 (60%)	30 (23%)	0
	Asp67Asn	150 (38%)	126 (55%)	24 (19%)	0
	Lys70Arg	125 (32%)	81 (35%)	43 (34%)	1 (3%)
	Leu210Trp	118 (30%)	110 (48%)	8 (6%)	0
	Thr215Phe	97 (25%)	75 (33%)	22 (17%)	0
	Lys219Gln	66 (17%)	42 (18%)	24 (19%)	0
	Lys219Glu	44 (11%)	40 (17%)	4 (2%)	0
	Lys65Arg	29 (7%)	25 (11%)	4 (2%)	0
Most common NRTI mutation pattern					
	41Leu, 184Val, 210Trp, 215Tyr	40 (11%)	40 (17%)	0	0
	184Val	38 (10%)	1	27	10
	41Leu, 67Asn, 184Val, 210Trp, 215Tyr	26 (7%)	26	0	0
	67Asn, 70Arg, 184Val, 219Gln	18 (5%)	1	17	0
	184Val, 215Tyr	15 (4%)	0	15	0
First-line NRTIs used					
	Zidovudine	275 (70%)	177 (80%)	86 (67%)	12 (36%)
	Tenofovir	39 (10%)	19 (8%)	16 (13%)	4 (12%)
Initial second-line NRTIs prescribed					
	Tenofovir, lamivudine/emtricitabine	279 (71%)	159 (69%)	105 (82%)	15 (45%)
	Tenofovir, zidovudine, lamivudine/emtricitabine*	36 (9%)	17 (7%)	8 (6%)	11 (33%)
	Abacavir, didanosine	51 (13%)	40 (17%)	5 (4%)	6 (18%)
	Abacavir, lamivudine/emtricitabine	12 (3%)	9 (4%)	2 (2%)	1 (3%)
	Zidovudine, lamivudine/emtricitabine	10 (3%)	2 (1%)	8 (6%)	0
	Didanosine, lamivudine/emtricitabine	2 (1%)	2 (1%)	0	0
	Zidovudine, didanosine	1 (<1%)	1 (<1%)	0	0

Data are median (IQR) or median (IQR; range), and number of patients and % of those with baseline genotypes available in each group. Predicted-active NRTIs are prescribed NRTIs with no more than low-level resistance on baseline genotype. ART=antiretroviral therapy. NRTI=nucleoside reverse-transcriptase inhibitor. GSS=genotypic susceptibility score. TAM=thymidine analogue mutation.

* An NRTI regimen of three NRTIs (tenofovir, zidovudine, and lamivudine) was initially prescribed in Malawi, but this was reduced to two NRTIs (usually tenofovir and lamivudine) after a median of 45 weeks from randomisation when national guidelines changed.

At week 144, 176 (89%) of 198 prescribed a second-line protease inhibitor and NRTI regimen containing no predicted-active NRTIs had viral suppression (viral load <400 copies per mL) compared with 312 (81%) of 383 in the protease inhibitor and raltegravir group (risk difference 7·4%; 95% CI 1·6 to 13·3; p=0·02). At week 96, 181 (88%) of 205 prescribed a regimen with no predicted-active NRTIs had viral suppression compared with 233 (61%) of 380 in the protease inhibitor monotherapy group (27·0%; 20·4 to 33·6; p<0·0001; figure). At week 144, 95 (85%) of 112 prescribed a regimen with one predicted-active NRTI had viral load suppression compared with 176 (89%) of 198 prescribed a regimen with no predicted-active NRTIs (−4·1%; −12·0 to 3·9; p=0·30). Furthermore, 20 (77%) of 26 with two to three predicted-active NRTIs had viral suppression (−12·0%; −28·7 to 4·8%; p=0·08 compared with no predicted-active NRTIs). Across the whole follow-up period, greater predicted activity of prescribed NRTIs was associated with worse viral load suppression (global generalised estimating equation p=0·0004; figure). A similar pattern of responses for all study weeks was seen with predicted activity of prescribed NRTIs calculated by genotypic susceptibility score (p=0·003; figure), and in analyses including only those failing on tenofovir first-line (p<0·0001), with viral load thresholds of less than 50 copies per mL (same rank, but difference attenuated, p=0·4; appendix p 3), or less than 1000 copies per mL (p=0·003; appendix p 4), and excluding patients in Malawi taking three NRTIs (p=0·03; appendix p 5).

**Figure:**
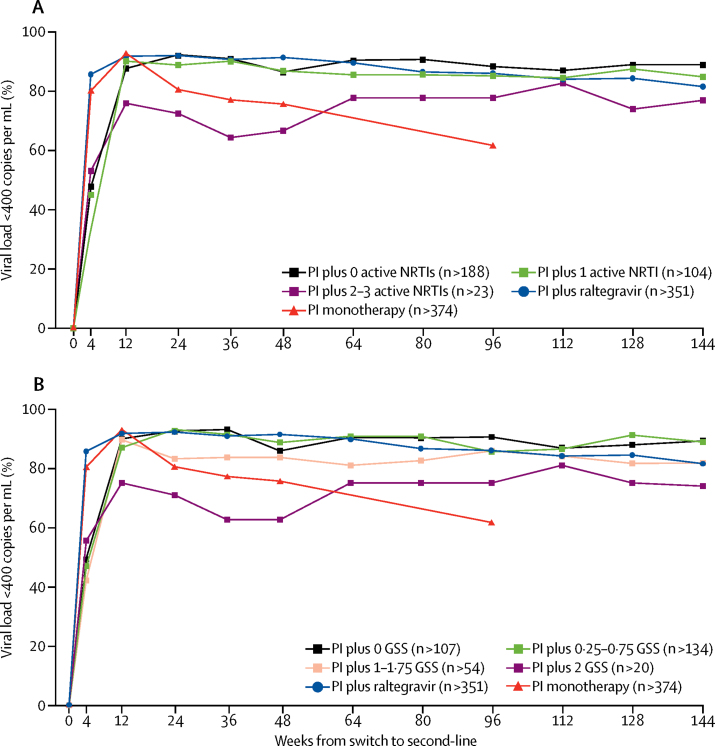
Viral load suppression by second-line regimen Suppression defined as <400 copies per mL. Predicted active NRTIs are prescribed NRTIs with no more than low-level resistance on baseline genotype (A) By number of predicted-active NRTIs; global p<0·0001; within PI and NRTI group, global p<0·0004. (B) By genotypic susceptibility score; global p<0·0001; within PI and NRTI group, global p=0·003. GSS=genotypic susceptibility score. NRTI=nucleoside reverse-transcriptase inhibitor. n=minimum number of viral load values available at any follow-up timepoint in each group. PI=protease inhibitor.

In the protease inhibitor and NRTI group, increased viral load or reduced CD4 cell count at baseline, being unemployed or a student, working more hours if employed, and reporting being non-adherent at more visits were all independently associated with reduced probability of a viral load of less than 400 copies per mL at week 144 (table 2). Adjusting for these factors, increased genotypic susceptibility score remained associated with reduced probability of a viral load of less than 400 copies per mL (p=0·001); the effect remained (p=0·004) after restricting analysis to those with a genotypic susceptibility score of less than 2 (appendix p 8). Adjusting for these factors and genotypic susceptibility score, there was no evidence of an association between any individual mutation, TAM1, or TAM2 and a viral load of less than 400 copies per mL (p>0·05, data not shown). Results were very similar including all factors with p<0·2 on univariable analysis without backwards elimination, and using backward elimination with exit p>0·2 (appendix p 8).

**Table 2:** Multivariable model for viral load <400 copies per mL at week 144 in protease inhibitor and nucleoside reverse-transcriptase inhibitor group

	**Unadjusted odds ratio (95% CI)**	**p value**	**Adjusted odds ratio (95% CI)**	**p value**
Genomic susceptibility score of second-line regimen (per 0·5 higher)	0·78 (0·61–0·99)	0·04	0·61 (0·46–0·81)	0·001
Proportion of non-adherent visits (per 10% higher)*	0·66 (0·55–0·79)	<0·0001	0·61 (0·49–0·77)	<0·0001
Unemployed or student *vs* employed	0·39 (0·21–0·72)	0·003	0·22 (0·07–0·63)	0·005
Hours worked by employed and students (per 10 h higher)	1·03 (0·92–1·17)	0·6	0·83 (0·70–0·99)	0·04
Baseline viral load per doubling	0·82 (0·70–0·95)	0·01	0·80 (0·67–0·97)	0·02
Baseline CD4 cell count per doubling	1·24 (1·02–1·50)	0·03	1·33 (1·04–1·71)	0·02

n=317, excluding those with missing week 144 viral load, baseline genotype or baseline employment status. Baseline refers to switch to second-line therapy (enrolment into the trial). Univariable analyses are in the appendix. Adjusted odds ratio adjusted for the factors given in the table. Factors not selected (p>0·05): sex, age, viral subtype, years on first-line antiretroviral treatment, diabetes, family history of cardiovascular disease, previous CNS disease, previous tuberculosis, smoking, alcohol consumption, household income, food availability, years of education, estimated glomerular filtration rate, haemoglobin, glucose, presence of individual mutations in the baseline genotype (where >10% prevalence), presence of combinations of 2 mutations in the baseline genotype (where >10% prevalence).

* Scheduled visit that was either missed or where the participant self-reported missing pills since the last visit.

## Discussion

We found that NRTIs predicted by resistance testing to contribute limited or no antiviral activity to a protease inhibitor-containing second-line regimen still had substantial virological effect, at least as good as that obtained from a fully active drug from a new class (raltegravir). This suggests that in the context of a switch from a first-line NNRTI-based to a second-line protease-inhibitor-based regimen, predictions based on genotypic resistance testing might overestimate the detrimental effect of cross-resistance on the activity of NRTIs. Prediction of clinical outcomes from genotypic resistance tests is challenging,^10^, ^11^ and algorithms might be particularly poor at predicting the combined effects of NRTI mutations that could sometimes be antagonistic.

In addition to our finding of substantial efficacy of NRTIs that had limited or no predicted activity, the paradoxical association we found between greater predicted NRTI activity and worse viral load suppression also contrasts with standard assumptions underlying resistance. Our findings are consistent with other second-line studies that have also found an absent or paradoxical association between NRTI resistance and outcomes,^12^, ^13^, ^14^, ^15^, ^16^, ^17^ but several features of our study (especially the prospectively collected adherence data and availability of two comparison groups) allow for more robust interpretation. The inferior viral suppression observed in those individuals with two or more fully active NRTIs in the prescribed regimen is probably explained by treatment adherence: this very small group of patients had no resistance mutations detected at baseline and probably comprised those who concealed complete non-adherence at study entry and continued to have poor adherence during second-line. The high viral load suppression in those taking NRTIs with limited or no predicted activity is probably not explained by residual confounding by adherence, elevated viral load, or other baseline factors associated with resistance because the effects persisted after adjustment. Furthermore, the randomised comparison of the entire protease inhibitor and NRTI group versus the protease inhibitor monotherapy group (ie, with all confounding removed) showed viral load suppression was increased in the protease inhibitor and NRTI group overall, which can only be because of the presence of ongoing NRTI activity.^7^ Our interpretation is further supported by a randomised comparison of protease inhibitor monotherapy versus protease inhibitor plus lamivudine as maintenance in second-line therapy that showed clear additional contribution of lamivudine despite almost universal lamivudine resistance.^18^

The observed NRTI effect might, in part, arise from resistance mutations such as Met184Val and Lys65Arg affecting viral replicative capacity, with effects increasing with more mutations.^19^, ^20^, ^21^ The effect of resistance mutations on viral replication fidelity might also protect the protease inhibitor by limiting the development of new mutations to escape drug pressure, thus increasing regimen durability.^22^, ^23^ Pharmacokinetic considerations might enhance these benefits; NRTIs, such as tenofovir and lamivudine, have long intracellular half-lives that might help to maintain viral suppression when protease inhibitor levels are low (with late or occasional missed doses) and this effect might be independent of predicted activity. This potential effect could explain why we observed that the NRTIs were capable of matching the suppression obtained with raltegravir (a drug with a relatively short intracellular half-life) in combination with a protease inhibitor.

The strengths of our study include large numbers of patients, long follow-up, high retention, regular viral load testing at predetermined timepoints, centralised viral load and resistance testing, and the availability of two relevant treatment comparison groups (especially the protease inhibitor monotherapy group) that enable the contribution of NRTIs to the regimen to be delineated. Although patients were participating in a clinical trial, the trial eligibility criteria were kept broad and we followed the widely used approach of clinical and CD4 cell count monitoring (targeted viral load testing was done to confirm treatment failure before changing therapy, but there was no real-time viral load testing during the trial). The results are therefore probably generalisable to patients failing first-line therapy in programmes that follow the public health approach to ART delivery.

The main limitation of our study is that most patients were taking zidovudine-based first-line regimens; whereas, contemporary WHO guidelines recommend tenofovir-based first-line ART. The similar results seen in the analysis of the subgroup failing on first-line tenofovir, however, support the generalisability of our findings. The protease inhibitor was standardised in this trial to ritonavir-boosted lopinavir, which is recommended as a preferred protease inhibitor in WHO guidelines^3^ and remains widely used in treatment programmes following the WHO public health approach. Whether NRTIs with limited or no predicted activity would contribute in a similar way to a regimen with atazanavir (WHO preferred protease inhibitor) or darunavir (alternative protease inhibitor) is unclear, although this seems probable at least for darunavir on the basis of its activity with NRTI resistance in other second-line studies.^18^, ^24^ We analysed our data with the Stanford algorithm, the most widely used. Although alternative algorithms might give slightly different weight to individual resistance mutations, they are based on the same mutations and would probably provide similar outcome predictions.^10^ Patients had non-subtype-B virus, whereas the reference algorithms in the Stanford database are for subtype-B virus. Mutations conferring NRTI drug resistance differ little between subtypes, however, and do not seem to affect outcomes.^25^, ^26^ A cohort study^17^ in a subtype-B population drew similar conclusions with respect to predicted NRTI activity and outcomes. For our binary classification of NRTI drugs as active or inactive we used a cutoff (NRTI considered active if no more than low-level resistance) that corresponds to the way many clinicians interpret resistance tests. Although some researchers might prefer alternative cutoffs, we found the same results with an analysis based on genotypic susceptibility score that gives more discrimination at intermediate values of resistance, and on a linear regression analysis of the genotypic susceptibility score that dispensed entirely with cutoffs. Furthermore, arguments on cutoffs are irrelevant for the group in which there was high-level resistance to both NRTI drugs selected (in which genotypic susceptibility score=0). In this situation, most would expect little benefit from the prescribed drugs and yet there was clear evidence of a major contribution to regimen activity.

Our findings are relevant to discussions about the merit of introducing routine resistance testing into the public health approach to ART for patients switching to the standard protease inhibitor-based second-line regimen. Although genotypic resistance testing is recommended in treatment guidelines for individualised therapy and widely used by clinicians in high-income countries for ART drug selection at treatment failure,^4^, ^5^, ^6^ the randomised controlled trials on which this practice is based generally showed modest short-term benefit and, of particular relevance to the present discussion, enrolled mainly patients who had failed on protease inhibitor-based regimens.^27^ None of these trials specifically examined the benefit of genotypic resistance testing in patients not previously treated with a protease inhibitor switching from a failing NNRTI-based first-line regimen to a protease inhibitor-based second-line regimen. The lack of association between predicted NRTI activity and outcomes shown in our study and other observational studies of protease inhibitor-based second-line therapy supports WHO treatment guidelines that do not recommend resistance testing at switch to second-line ART in the public health approach.^3^ The findings also suggest that NRTI selection might best be based on minimising toxicity and maximising tolerability in view of the effect of these on adherence. Introducing resistance testing in programme settings could theoretically provide additional benefit by identifying patients without resistance mutations who might be managed with more adherence counselling rather than switching to second-line. Our data suggest the effect of this would also be limited, however, because such patients were uncommon in our population (less than 2% lacked either an NRTI or NNRTI mutation).^22^ The proportion without resistance mutations detected at failure might be higher in programmes that succeed in implementing the WHO recommendation of annual viral load testing, but in some of these cases the resistance mutations might be present but archived (ie, would re-emerge upon resumption of regular therapy). Furthermore, such patients might not be responsive to additional adherence counselling: in our trial we provided universal adherence counselling at first-line failure and during second-line, but such patients still had inferior outcomes on second-line therapy. The NRTI drug class will probably remain in widespread use for millions of people in treatment programmes that use the public health approach (and elsewhere). Our results should stimulate further work to validate resistance-testing algorithms against NRTI clinical response in second-line protease inhibitor-based therapy and in other drug regimens. More generally, our findings emphasise the need for critical thinking around the benefits to be gained, if any, before new elements of care are introduced into the public health approach, even if they are considered as standard practice in high-income settings.
