# Supplementary material for: Nucleoside reverse-transcriptase inhibitor cross-resistance and outcomes from second-line antiretroviral therapy in the public health approach: an observational analysis within the randomised, open-label, EARNEST trial
Source: Lancet HIV. 2017 May 8;4(8):e341–8. doi: 10.1016/S2352-3018(17)30065-6 (PMC5555436; doi:10.1016/S2352-3018(17)30065-6)
Supplement: Supplementary appendix [file mmc1.pdf]

THE LANCET
HIV

Supplementary appendix

This appendix formed part of the original submission and has been peer reviewed. We post it as supplied by the authors.

Supplement to: Paton N I, Kityo C, Thompson J, et al. Nucleoside reverse-transcriptase inhibitor cross-resistance and outcomes from second-line antiretroviral therapy in the public health approach: an observational analysis within the randomised, open-label, EARNEST trial. *Lancet HIV* 2017; published online May 8. http://dx.doi.org/10.1016/S2352-3018(17)30065-6.

SUPPLEMENTARY MATERIALS

Members of the EARNEST Trial Team are:

Participating Sites

Uganda

JCRC Kampala (African trial co-ordinating centre; 231) E Agweng, P Awio, G Bakeinyaga, C Isabirye, U Kabuga, S Kasuswa, M Katuramu, C Kityo, F Kiweewa, H Kyomugisha, E Lutalo, P Mugyenyi, D Mulima, H Musana, G Musitwa, V Musiime, M Ndigendawan, H Namata, J Nkalubo, P Ocitti Labejja, P Okello, P Olal, G Pimundu, P Segonga, F Ssali, Z Tamale, D Tumukunde, W Namala, R Byaruhanga, J Kayiwa, J Tukamushaba, S Abunyang, D Eram, O Denis, R Lwalanda, L Mugarura, J Namusanje, I Nankya, E Ndashimye, E Nabulime, D Mulima, O Senfuma.
IDI, Kampala (216): G Bihabwa, E Buluma, P Easterbrook, A Elbireer, A Kambugu, D Kamya, M Katwere, R Kiggundu, C Komujuni, E Laker, E Lubwama, I Mambule, J Matovu, A Nakajubi, J Nakku, R Nalumenya, L Namuyimbwa, F Semitala, B Wandera, J Wanyama

JCRC, Mbarara (97): H Mugerwa, A Lugemwa, E Ninsiima, T Ssenkindu, S Mwebe, L Atwine, H William, C Katemba, S Abunyang, M Acaku, P Ssebutinde, H Kitizo, J Kukundakwe, M Naluguza, K Ssegawa, Namayanja, F Nsibuka, P Tuhirirwe, M Fortunate

JCRC Fort Portal (66): J Acen, J Achidri, A Amone, M. Chamai, J Ditai, M Kemigisa, M Kiconco, C Matama, D Mbanza, F Nambaziira, M Owor Odoi, A Rweyora, G. Tumwebaze

San Raphael of St Francis Hospital, Nsambya (48): H Kalanzi, J Katabaazi, A Kiyingi, M Mbidde, M. Mugenyi, R Mwebaze, P Okong, I Senoga

JCRC Mbale (47): M Abwola, D Baliruno, J Bwomezi, A Kasede, M Mudoola, R Namisi, F Ssennono, S Tuhirwe

JCRC Gulu (43): G Abongomera, G Amone, J Abach, I Aciro, B Arach, P Kidega, J Omongin, E Ocung, W Odong, A Philliam

JCRC Kabale (33): H Alima, B Ahimbisibwe, E Atuhaire, F Atukunda, G Bekusike, A Bulegyeya, D. Kahatano, S Kamukama, J Kyoshabire, A Nassali, A Mbonye, T M Naturinda, Ndukukire, A Nshabohurira, H. Ntawiha, A Rogers, M Tibyasa;

JCRC Kakira (31): S. Kiirya, D. Atwongyeire, A. Nankya, C. Draleku, D. Nakiboneka, D. Odoch, L. Lakidi, R. Ruganda, R. Abiriga, M. Mulindwa, F. Balmoi, S. Kafuma, E. Moriku

Zimbabwe

University of Zimbabwe Clinical Research Centre, Harare (265): J Hakim, A Reid, E Chidziva, G Musoro, C Warambwa, G Tinago, S Mutsai, M Phiri, S Mudzingwa, T Bafana, V Masore, C Moyo, R Nhema, S Chitongo

Malawi

Department of Medicine, University of Malawi College of Medicine and the Malawi-Liverpool-Wellcome Trust Clinical Research Programme, University of Malawi College of Medicine (92): Robert Heyderman,

Lucky Kabanga, Symon Kaunda, Aubrey Kudzala, Linly Lifa, Jane Mallewa, Mike Moore, Chrissie Mtali, George Musowa, Grace Mwimaniwa, Rosemary Sikwese, Joep van Oosterhout, Milton Ziwoya

Mzuzu Central Hospital, Mzuzu (19): H Chimbaka. B Chitete, S Kamanga, T Kayinga E Makwakwa, R Mbiya, M Mlenga, T Mphande, C Mtika, G Mushani, O Ndhlovu, M Ngonga, I Nkhana, R Nyirenda

Kenya

Moi Teaching and Referral Hospital (52): P Cheruiyot, C Kwobah, W Lokitala Ekiru, M Mokaya, A Mudogo, A Nzioka, A Siika, M Tanui, S Wachira, K Wools-Kaloustian

Zambia

University Teaching Hospital (37): P Alipalli, E Chikatula, J Kipaila, I Kunda, S Lakhi, J Malama, W Mufwambi, L Mulenga, P Mwaba, E Mwamba, A Mweemba, M Namfukwe

The Aids Support Organisation (TASO), Uganda: E Kerukadho, B Ngwatu, J Birungi

MRC Clinical Trials Unit: N Paton, J Boles, A Burke, L Castle, S Ghuman, L Kendall, A Hoppe, S Tebbs, M Thomason, J Thompson, S Walker, J Whittle, H Wilkes, N Young, M Spyer

Monitors: C Kapuya, F Kyomuhendo, D Kyakundi, N Mkandawire, S Mulambo, S Senyonjo

Clinical Expert Review Committee: B Angus, A Arenas-Pinto, A Palfreeman, F Post, D Ishola

European Collaborators:

J Arribas (Hospital La Paz, Madrid, Spain), R Colebunders (Institute of Tropical Medicine, Antwerp, Belgium), M Floridia (ISS, Italy), M Giuliano (ISS, Italy), P Mallon (University College Dublin, Ireland), P Walsh (University College Dublin, Ireland), M De Rosa (CINECA, Italy), E Rinaldi (CINECA, Italy)

Trial Steering Committee: I Weller (Chair), C Gilks, J Hakim, A Kangewende, S Lakhi, E Luyirika, F Miiro, P Mwamba, P Mugyenyi, S Ojoo, N Paton, S Phiri, J van Oosterhout, A Siika, S Walker, A Wapakabulo,

Data Monitoring Committee: T Peto (Chair), N French, J Matenga

Pharmaceutical companies: G Cloherty, J van Wyk, M Norton, S Lehrman, P Lamba, K Malik, J Rooney, W Snowden, J Villacian

SUPPLEMENTARY FIGURES

Supplementary Figure 1: Viral load suppression <50 c/ml by number of predicted-active NRTIs in prescribed second-line regimen

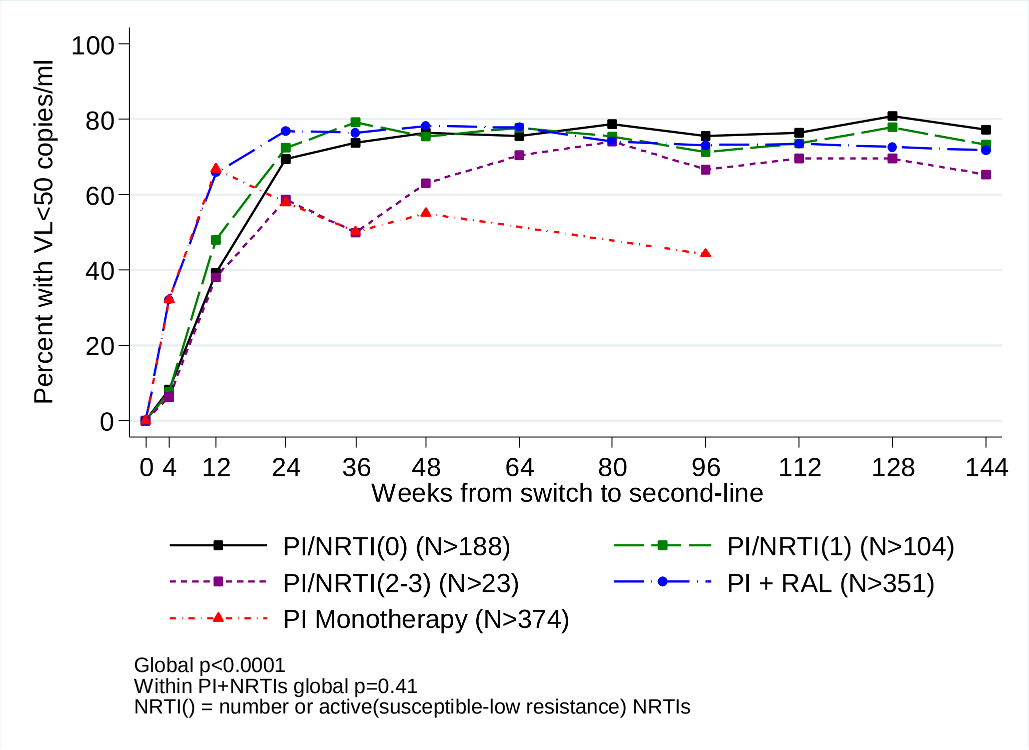

Supplementary Figure 2: Viral load suppression <1000 c/ml by number of predicted-active NRTIs in prescribed second-line regimen

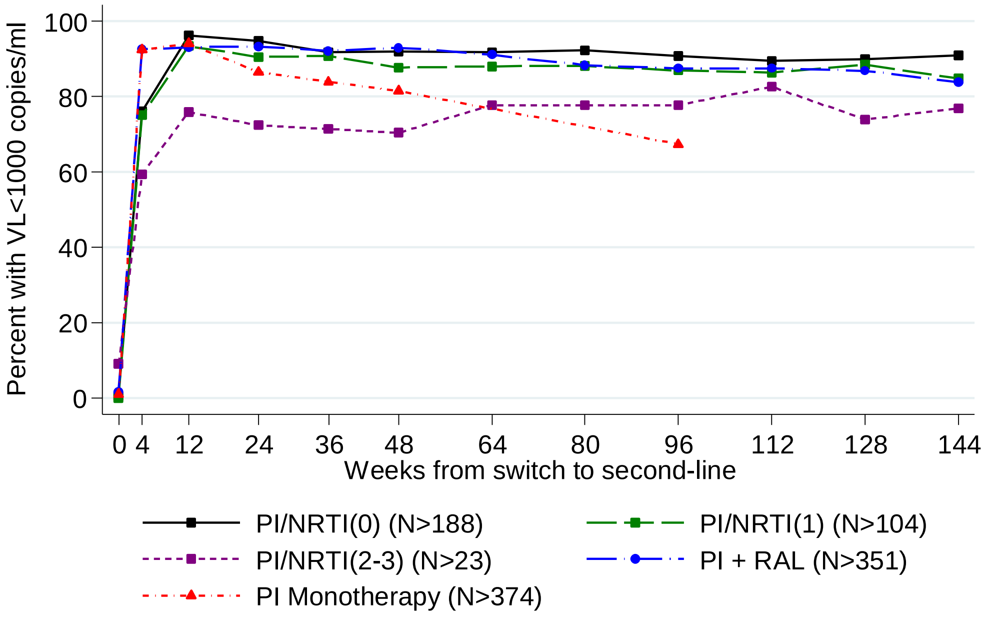

Global p<0.0001
Within PI+NRTIs global p=0.003
NRTI() = number or active(susceptible-low resistance) NRTIs

Supplementary Figure 3:Viral load suppression <400 copies/ml by number of predicted-active NRTIs in prescribed second-line regimen, excluding Malawi

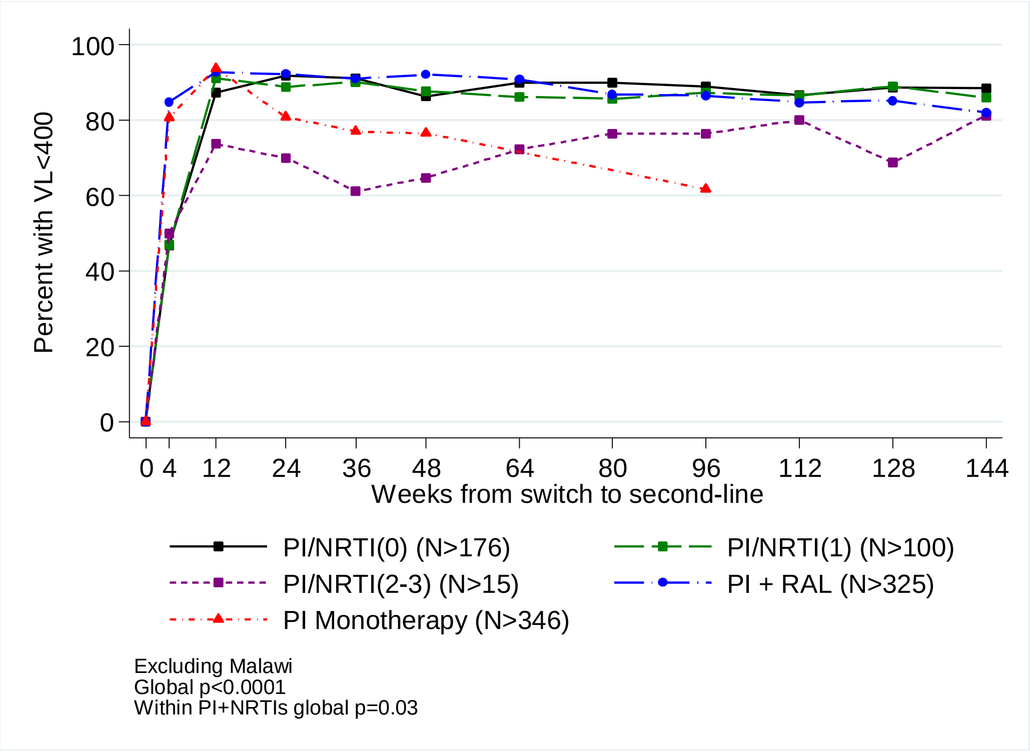

Supplementary Table 1: Crude associations with viral load suppression <400 copies/ml

Characteristic	Total N=367	VL<400 copies/ml N=317	VL>400 copies/ml N=50	p value
Genotypic sensitivity score (GSS)	0.25(0-0.75)	0.25(0-0.5)	0.5(0-1)	0.04
Number of ART drugs with less than intermediate resistance				0.19
0	198/336(59%)	176/291(60%)	22/45(49%)	
1	112/336(33%)	95/291(33%)	17/45(38%)	
2	26/336(8%)	20/291(7%)	6/45(13%)	
Age	37(31-44)	37(31-44)	35(30-42)	0.15
Female sex	228/367(62%)	198/317(62%)	30/50(60%)	0.74
Subtype				0.31
A	138/343(40%)	116/293(40%)	22/50(44%)	
C	108/343(31%)	94/293(32%)	14/50(28%)	
D	79/343(23%)	70/293(24%)	9/50(18%)	
other	18/343(5%)	13/293(4%)	5/50(10%)	
Proportion of visits missed or reporting non-adherence	9%(5%-23%)	9%(5%-18%)	23%(9%-41%)	<0.0001
Years on first-line ART	4(3-5)	4(3-6)	4(3-5)	0.07
Ever smoked	50/366(14%)	43/316(14%)	7/50(14%)	0.94
Ever drank alcohol	45/320(14%)	40/278(14%)	5/42(12%)	0.67
Job status				0.002
Employed	236/366(64%)	214/317(68%)	22/49(45%)	
Student	29/366(8%)	20/317(6%)	9/49(18%)	
Unemployed	101/366(28%)	83/317(26%)	18/49(37%)	
Work hours if employed	25(0-48)	25(0-48)	12(0-40)	0.59
Household income (USD/month)				0.09
<50	172/317(54%)	145/275(53%)	27/42(64%)	
50-200	94/317(30%)	81/275(29%)	13/42(31%)	
>200	51/317(16%)	49/275(18%)	2/42(5%)	
Sufficient food for regular meals	239/367(65%)	208/317(66%)	31/50(62%)	0.62
Years of education	9(7-12)	9(7-12)	9(7-12)	0.71
Diabetes	6/367(2%)	6/317(2%)	0/50(0%)	0.33
TB before switch to second-line	91/367(25%)	78/317(25%)	13/50(26%)	0.83
Family history of cardiovascular disease	50/281(18%)	44/248(18%)	6/33(18%)	0.95
Baseline log_{10} viral load	5(4-5)	5(4-5)	5(5-5%)	0.01
Baseline CD4	73(32-140)	74(36-143)	56(20-107)	0.16

Characteristic	Total N=367	VL<400 copies/ml N=317	VL>400 copies/ml N=50	p value
Baseline haemoglobin	12(11-13)	12(11-13)	11(10-14)	0.32
Baseline glomerular filtration rate (Cockcroft-Gault formula)	114(88-135)	113(88-135)	121(92-148)	0.20
Baseline blood glucose	4(4-5)	4(4-5)	4(4-5)	0.32

Supplementary Table 2: Multivariable associations with viral suppression <400 copies/ml within the subgroup of patients with GSS<2

	Adjusted odds ratio (95% CI)	P value
GSS of second line regimen (per 0.5 higher)	0.42 (0.24, 0.75)	0.004
Proportion of non-adherent visits (per 10% higher)	0.59 (0.46, 0.76)	0.0001
Unemployed or student vs employed	0.22 (0.07, 0.74)	0.01
Hours worked by employed and students (per 10 hours higher)	0.82 (0.68, 1.00)	0.05
Baseline viral load per doubling	0.73 (0.58, 0.92)	0.009
Baseline CD4 per doubling	1.42 (1.07, 1.89)	0.02

N=296

Supplementary Table 3: Multivariable associations with viral suppression <400 copies/ml, including all factors with p<0.2 in univariable analyses

	Adjusted odds ratio (95% CI)	p value
GSS of second line regimen (per 0.5 higher)	0.67 (0.50, 0.91)	0.02
Proportion of non-adherent visits (per 10% higher)	0.66 (0.52, 0.82)	0.0003
Unemployed or student vs employed	0.33 (0.15, 0.75)	0.008
Household income USD/month (vs <50)*		
50-200	0.80 (0.33, 1.93)	0.31
>200	2.94 (0.61, 14.18)	
Age (per 10 year increase)*	0.98 (0.95, 1.02)	0.29
Years on first line ART*	1.10 (0.91, 1.33)	0.35
Baseline viral load per doubling	0.77 (0.64, 0.93)	0.007
Baseline CD4 per doubling	1.31 (1.01, 1.72)	0.05

* factor not included in Table 2 model based on backwards elimination with exit p>0.05
N=290

Supplementary Table 4: Multivariable associations with viral suppression <400 copies/ml based on backward selection exit p>0.2

	Adjusted odds ratio (95% CI)	P value
GSS of second line regimen (per 0.5 higher)	0.61 (0.46, 0.81)	0.001
Proportion of non-adherent visits (per 10% higher)	0.61 (0.49, 0.77)	<0.0001
Unemployed or student vs employed	0.22 (0.07, 0.63)	0.005
Hours worked by employed and students (per 10 hours higher)	0.83 (0.70, 0.99)	0.04
Baseline viral load per doubling	0.80 (0.67, 0.97)	0.02
Baseline CD4 per doubling	1.33 (1.04, 1.71)	0.02

N=317. Identical model selected as Table 2 model based on backwards elimination with exit p>0.20
